# High-Yield Production of Receptor Binding Domain of SARS-CoV-2 Linked to Bacterial Flagellin in Plants Using Self-Replicating Viral Vector pEff

**DOI:** 10.3390/plants10122682

**Published:** 2021-12-06

**Authors:** Eugenia S. Mardanova, Roman Y. Kotlyarov, Nikolai V. Ravin

**Affiliations:** Institute of Bioengineering, Research Center of Biotechnology of the Russian Academy of Sciences, 119071 Moscow, Russia; mardanovaes@mail.ru (E.S.M.); bercut@gmail.com (R.Y.K.)

**Keywords:** coronavirus, SARS-CoV-2, plant, expression, recombinant vaccine, flagellin

## Abstract

The development of recombinant vaccines against SARS-CoV-2 is required to eliminate the COVID-19 pandemic. We reported the expression of a recombinant protein Flg-RBD comprising receptor binding domain of SARS-CoV-2 spike glycoprotein (RBD) fused to flagellin of *Salmonella typhimurium* (Flg), known as mucosal adjuvant, in *Nicotiana benthamiana* plants. The fusion protein, targeted to the cytosol, was transiently expressed using the self-replicating vector pEff based on potato virus X genome. The recombinant protein Flg-RBD was expressed at the level of about 110–140 μg per gram of fresh leaf tissue and was found to be insoluble. The fusion protein was purified using metal affinity chromatography under denaturing conditions. To increase the yield of Flg-RBD, the flow-through fraction obtained after loading of the protein sample on the Ni-NTA resin was re-loaded on the sorbent. The yield of Flg-RBD after purification reached about 100 μg per gram of fresh leaf tissue and the purified protein remained soluble after dialysis. The control flagellin was expressed in a soluble form and its yield after purification was about 300 μg per gram of fresh leaf biomass. Plant-produced Flg-RBD protein could be further used for the development of intranasal recombinant mucosal vaccines against COVID-19.

## 1. Introduction

The emergence of new pathogenic strains of viruses is a constant threat to human health throughout the world. The Severe acute respiratory syndrome coronavirus 2 (SARS-CoV-2) is a novel coronavirus that caused an ongoing pandemic in the human population. An outbreak of coronavirus disease 2019 (COVID-19) was reported in December 2019 in the city of Wuhan, Hubei province of China. The COVID-19 pandemic has rapidly spread all over the world and has put global public health at high risk [1]. Nowadays, the acronym COVID-19 is one of the most recognizable in the world.

The coronaviruses are enveloped positive-sense single-stranded RNA viruses [2]. Coronaviruses form spherical particles with a diameter of 100–160 nm. One-third of the genome (~30 kb) of SARS-CoV2 encodes the structural proteins of the virus: Spike glycoprotein (S), nucleocapsid protein (N), small envelop protein (E), and matrix protein (M). The S protein of the SARS-CoV-2 coronavirus forms a trimer on the surface of the viral particle and forms a viral spike, which plays a crucial role in the cell entrance process. The amino acid sequence of the S protein contains a signal for proteolytic cleavage into S1 and S2 fragments, but they remain linked through non-covalent interactions [3]. S1 and S2 subunits are responsible for binding to the cell receptor and fusion with the membrane, respectively [4,5]. In the structure of the S-protein obtained using cryo-electron microscopy [3,6], it is possible to distinguish the receptor-binding domain (RBD, amino acids 319–541), which binds directly to the receptor of the angiotensin-converting enzyme 2 (ACE2) [7]. RBD is critically important for the binding of the virus to the host cell and therefore it is a promising antigen for vaccine development [3]. Most of the potent monoclonal antibodies to SARS-CoV-2 target the RBD [8]. An additional argument for choosing the RBD domain is that increased vaccine-mediated pressure on the virus stimulates the occurrence of mutations in the RBD area, which in turn reduces the affinity level of the RBD to the ACE2 receptor.

Recombinant proteins can be produced in different expression systems, including bacteria, yeast, plants, and mammalian cells. Numerous studies showed that plants could be a promising expression platform for the production of recombinant protein. Cultivation of plants does not require complex equipment and could be easily scaled up. Plants along with animal expression systems can perform post-translation modifications while providing safety of products resulting from the absence of pathogens common to plants and animals [9]. Nevertheless, the plant-based expression has also several shortcomings such as (i) low productivity compared to bacterial and mammalian cell cultures [10,11] and (ii) relatively high downstream purification costs, since plant cells need to be lysed to obtain the target protein, while mammalian cells can secrete the target protein to the medium [10,12,13]. Recombinant proteins can be produced in transgenic plants, but their development requires a long time (several months, depending on plant species) and the yield of recombinant protein is usually low (less than 10 μg per gram of fresh biomass) [14]. A more promising alternative is the use of transient expression systems based on plant viral vectors which allow the production of recombinant proteins at a high level (up to 5 mg per gram of plant biomass) within several days [15,16,17,18,19,20,21,22,23,24].

The COVID-19 pandemic has encouraged research in the area of plant molecular farming [25,26,27]. A plant-based expression system could be a promising platform for the fast, cost-effective, and scalable production of SARS-CoV-2 proteins as candidate recombinant COVID-19 vaccines [25,27]. Significant progress in the expression of viral antigens in plants has been demonstrated by Medicago (Quebec City, QC, Canada). For the first time, this company has submitted a vaccine against seasonal influenza of plant origin, which has passed three stages of clinical trials, for consideration by regulatory authorities [28]. Medicago successfully produced in plants a virus-like particle (VLP) candidate vaccine carrying the SARS-CoV-2 S protein just 20 days after obtaining the gene [29]. IBio company has announced [30] that it is developing a plant-produced candidate recombinant subunit vaccine based on fragments of SARS-CoV-2 S protein fused to a carrier protein LickM, derived from β-1, 3-1, 4-glucanase of *Clostridium thermocellum* which has previously been used with influenza hemagglutinin [31]. Patented lichenase booster molecule, LickM, as part of fusion provided increased solubility and expression, safety, robust immune response, long-lasting immunity [32]. Similarly, Kentucky BioProcessing has announced that it is developing a plant-produced recombinant protein subunit RBD-based vaccine and already started phase 1–2 clinical trials (NCT04473690). No detailed information on these recombinant candidate COVID-19 vaccines has been published [30]. The group of George Lomonossoff (John Iness Centre, Norwich, UK) showed that transient simultaneous expression of E, M, and S proteins in *Nicotiana benthamiana* leads to the formation of VLPs [30].

Recombinant protein-based vaccines can be designed as subunit vaccines comprised of purified immunogenic proteins or peptides derived from viruses. As a rule, subunit vaccines require adjuvants to stimulate the immune system because of their weak immunogenicity [33]. Alum salts, oil-in-water emulsions, liposomes, saponins, and other compounds can be used as adjuvants [34,35]. An alternative approach is the linking of the target antigen to a highly immunogenic carrier protein acting as an adjuvant. Many common adjuvants that activate innate immunity belong to a class of molecules known as pathogen-associated molecular patterns, including Toll-like receptors (TLRs) [33,36]. It has been reported in several studies that genetic fusion of the target antigen to bacterial flagellin, the ligand for TLR5, significantly increases the immunogenicity of the antigen and enhances protective properties [37,38]. It is important to note that flagellin is particularly potent as a mucosal adjuvant, opening the possibility of non-invasive delivery of vaccines, for example, by the intranasal route. The intranasal route of antigen administration mimics natural infection and induces both local and systemic immune responses. Local immunity is mediated by secretory immunoglobulin A. The ability of flagellin-based vaccines to induce an immune response in the nasal compartment is particularly important for COVID-19 vaccines since it is the first barrier that the virus breaches before dissemination to the lung. Another advantage of mucosal vaccines is low reactogenicity and the minimal risk of accidental infection with foreign pathogens in comparison with intramuscular preparations. The efficacy of flagellin as a mucosal adjuvant has been demonstrated in the development of a number of recombinant vaccines, including influenza A vaccines based on the M2e peptide [38,39,40,41].

The expression of the receptor-binding domain of SARS-CoV-2 in plants has already been reported. In one study using a geminiviral plant expression vector, the authors expressed his-tagged RBD comprising amino acid 318–617 with the highest expression level of 8 μg/g of fresh leaf tissue [42]. In the second research, the RBD was expressed using a non-replicating vector pEAQ [43]. It was found that the level of expression and yield of his-tagged RBD (319–591 aa) after purification was less than 10 μg/g of fresh leaf tissue, however, RBD-FLAG tagged protein was significantly better expressed in plants and the purification yield of RBD-FLAG was more than 20 μg/g of fresh leaf biomass [43]. In the third study, his-tagged RBD (319–541 aa) was expressed at the level of 92 μg/g of fresh leaf biomass with the purification yield of 6 μg/g of fresh leaf biomass. The authors used a tobacco mosaic virus-based expression vector. This recombinant RBD elicited humoral immunity in mice via induction of neutralizing antibodies [44].

In the present study, we used the pEff vector [45] based on potato virus X (PVX) to express recombinant SARS-CoV-2 proteins in plants. This self-replicating vector was previously used for the fast high-level production of different recombinant proteins in plants, up to 1 mg/g of fresh leaf biomass in the case of GFP [40,45,46,47]. A fusion protein comprising the receptor-binding domain of SARS-CoV2 protein S and flagellin of *Salmonella typhimurium* was successfully produced in *N. benthamiana* plants using the pEff expression system.

## 2. Results

**Figure 1:**
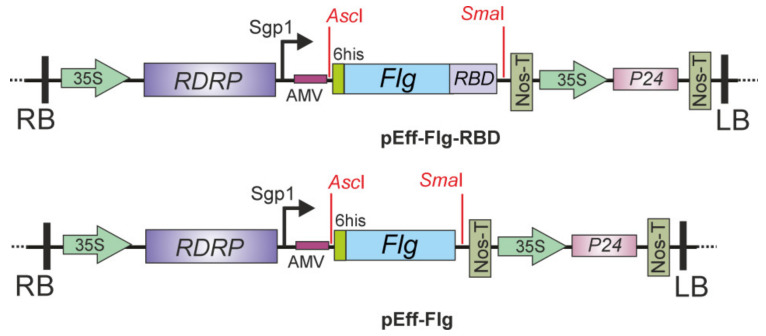
Scheme of the expression vectors pEff-Flg-RBD and pEff-Flg. RDRP, RNA-dependent RNA polymerase gene; Sgp1, the first promoter of subgenomic RNA of PVX; AMV, translational enhancer form alfalfa mosaic virus; 6 his, sequence encoding the hexahistidine tag; Flg, flagellin of *S. typhimurium*; RBD, the sequence of receptor-binding domain of SARS-CoV-2 from 319 to 524 aa; 35 S, promoter of the cauliflower mosaic virus RNA; Nos-T, terminator of the *A. tumefaciens* nopaline synthase gene; P 24, suppressor of silencing from grapevine leafroll-associated virus-2; RB and LB, the right and left borders of T-DNA region.

The pEff vector [45] was used to produce the recombinant fusion proteins in *N. benthamiana*. The RBD region spanning from amino acid 319 to 524 was genetically linked to the C-terminus of *Salmonella typhimurium* flagellin (Figure 1). Flagellin lacking RBD addition was used for comparison as a control. Both proteins, Flg-RBD and Flg, were engineered to comprise N-terminal hexahistidine tags to be purified using metal affinity chromatography. No sequences targeting recombinant proteins to a specific cellular compartment were used.

To obtain the viral vector pEff-Flg-RBD allowing expression of the Flg-RBD protein, the corresponding fusion gene was cloned in the pEff vector (Figure 1). Recombinant vector pEff-Flg for the expression of flagellin without RBD was constructed in a similar way (Figure 1). Vectors pEff-Flg-RBD and pEff-Flg were transfected into *A. tumefaciens* GV310 and the resulting agrobacterium cultures were used for the infiltration of the leaves of *N. benthamiana*.

**Figure 2:**
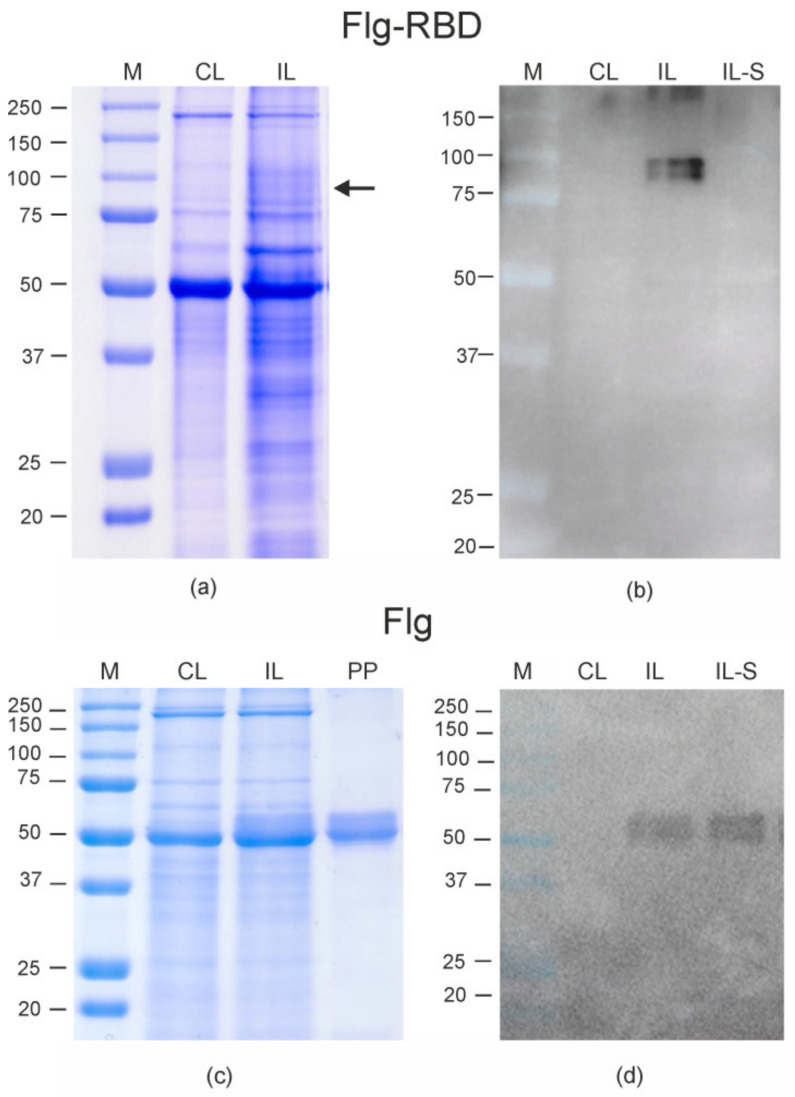
Expression of Flg-RBD (**a**,**b**) and Flg (**c**,**d**) proteins in *Nicotiana benthamiana* plants. Coomassie brilliant blue-stained gel (**a**,**c**) and Western blot (**b**,**d**) of proteins isolated from *N. benthamiana* plants. M, molecular weight marker (kD); CL, total proteins isolated from the non-infiltrated leaf; IL, total proteins isolated from leaf infiltrated with pEff-Flg-RBD (**a**,**b**) or pEff-Flg (**c**,**d**); IL-S, soluble fraction of proteins obtained from leaf infiltrated with pEff-Flg-RBD (**a**,**b**) or pEff-Flg (**c**,**d**); PP, purified Flg protein. Western blotting was performed using anti-flagellin antibodies. The position of Flg-RBD protein (calculated molecular weight 76 kD) in (**a**) is shown by an arrow.

Leaf tissues for isolation of protein samples were harvested four days after agroinfiltration when the level of the target protein expression was maximal (Supplementary File S1). In the following days, necrosis of leaf tissues in the infiltrated zones and degradation of Flg-RBD protein was observed. The necrosis can appear for several reasons, such as the toxicity of the protein or its individual fragments. An analysis of the total proteins and the soluble fraction by SDS-PAGE and Western blotting showed that Flg-RBD was successfully expressed but appeared to be fully insoluble (Figure 2). The expression level of Flg-RBD protein was about 110–140 μg/g of fresh leaf tissue.

Isolation of the recombinant Flg-RBD protein was performed using metal-affinity chromatography under denaturing conditions. To increase the yield of Flg-RBD, the flow-through fraction obtained after loading of the protein sample on the Ni-NTA resin was re-incubated with the sorbent. After elution from the sorbent, the purified protein samples were dialyzed against phosphate-buffered saline (PBS). After dialysis, the purified Flg-RBD protein remained soluble. The final yield of Flg-RBD after purification was about 90–100 µg/ g of fresh leaf biomass.

Expression of the control flagellin did not induce necrosis in agroinfiltrated plants. This protein was expressed more efficiently and was found to be soluble (Figure 2). The yield of Flg after purification was about 300 µg/ g of fresh leaf biomass.

**Figure 3:**
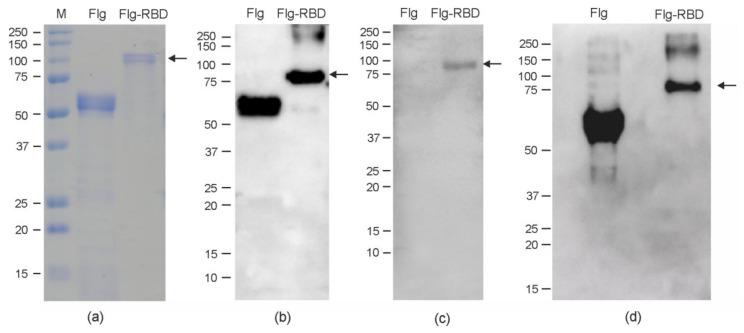
Purified Flg and Flg-RBD proteins were analyzed by SDS-PAGE and Western blotting. (**a**), Coomassie brilliant blue-stained SDS-PAGE gel of proteins isolated from *N. benthamiana*; western blot analysis with antibodies against hexahistidine tag (**b**), RBD (**c**), and flagellin (**d**). M, molecular weight marker (kD). The position of Flg-RBD protein is shown by an arrow.

Samples of purified Flg-RBD and Flg proteins were characterized using SDS-PAGE and Western blotting (Figure 3). The data shown in Figure 3 demonstrates that both proteins were purified. Both Flg-RBD and Flg were specifically revealed in Western blot analysis with the antibodies to flagellin and hexahistidine tag, while only Flg-RBD was detected with the monoclonal antibodies against the RBD.

We also attempted to use pEff vector for the expression of RBD without flagellin, but the low level of expression did not allow the detection of the protein in plant extracts using Western blotting and therefore the quantitative determination of the level of expression. Nevertheless, the yield of RBD protein after partial purification was about 13 μg/g of plant biomass (Supplementary File S2).

## 3. Discussion

The ongoing COVID-19 pandemic, caused by SARS-CoV-2, demands the development and fast large-scale production of cheap and effective vaccines against this pathogen. The purpose of our study was to develop a plant-based expression system for the production of the RBD of coronavirus SARS-CoV-2, linked to bacterial flagellin, known to be a potent mucosal adjuvant. For the expression of flagellin-based recombinant proteins, we used self-replicating viral vector pEff. Application of this vector allowed achieving the expression of Flg-RBD in plants at the level of 110–140 μg/g of fresh leaf biomass and the purification yield reached 100 μg/g. The expression of the RBD alone in plants has been reported in three publications with the highest purification yield of 20 μg/g of plant biomass [42,43,44]. Our attempts to use pEff vector for the expression of RBD gave similar yields.

The yield of Flg-RBD was however about three times lower than obtained for control empty flagellin and the fusion protein was produced in an insoluble form. The decreased expression level is likely related to the characteristics of the fusion partner, as previously reported for some other flagellin fusions [40]. Apparently, the lower yield and insolubility of Flg-RBD are not related to the hydrophobicity of the fusion partner, since the fraction of hydrophobic amino acid residues in RBD is even lower than in flagellin (39% vs. 48%). Perhaps the reason lies in the presence of seven cysteine residues in RBD, which can cause the formation of disulfide bonds and protein aggregation. It should also be noted that RBD expression induces necrosis in infiltrated leaves, as can be seen from Maharjan et al. [44]. RBD is likely toxic to plant cells and its fusion with flagellin weakens this effect, since RBD in the fusion protein may have a different conformation. Therefore, the fusion Flg-RBD protein can be obtained with a higher yield than RBD alone, but lower than control flagellin.

The efficiency of production of Flg-RBD in plants could be increased by several approaches, including the use of glycine-rich linkers for better spatial separation of flagellin and RBD, which may facilitate folding of the fusion protein, targeting of the fusion protein to the endoplasmic reticulum, expression in glycoengineered *N. benthamiana* plants [44]. Expression of the hybrid gene could be enhanced by the codon optimization for *N. benthamiana.* As recombinant proteins can be expressed in plants at a much higher level, in case of GFP up to 4 mg/g of plant biomass [9], a significant increase in the yield of Flg-RBD due to optimization of the expression system seems to be possible.

Overall, this research demonstrated that fast and relatively high-level expression of recombinant proteins comprising Flg and RBD of coronavirus SARS-CoV-2 in plants is feasible. Considering the prominent role of nasal mucosa in the transmission and the clinical progression of SARS-CoV-2, immunization using an intranasal vaccine based on RBD-flagellin fusion could be an effective strategy for immunoprophylaxis against SARS-CoV-2.

## 4. Materials and Methods

### 4.1. Expression Vector

The pEff vector contains the 5′-untranslated fragment of the PVX genome, the gene encoding an RNA-dependent RNA polymerase enabling the replication of the vector in a plant cell, promoter of the first viral subgenomic RNA, AMV translation enhancer (5′-nontranslated region of RNA 4 of the alfalfa mosaic virus), the *gfp* gene flanked by unique AscI and SmaI sites, the 3′ terminal part of the coat protein gene and the 3′-untranslated fragment of the PVX genome. This expression cassette is located downstream of the 35S promoter and followed by the Nos-T terminator. The pEff vector also contains the P 24 suppressor of silencing from grapevine leafroll-associated virus-2 under the control of the 35S promoter [45]. All these genetic elements are located within the T-DNA region of a binary vector that can be maintained both in *Escherichia coli* and *Agrobacterium tumefaciens*. Due to the presence of RNA-dependent RNA polymerase gene, the vector can self-replicate in a plant cell, which ensures a high level of expression of the target protein.

### 4.2. Gene Synthesis, Cloning and Construction of Expression Vectors

The RBD fragment of the S protein (from V319 to V524) of SARS-CoV-2 strain Wuhan-Hu-1 (GenBank QJE37812.1) was used as an antigen; the synthetic nucleotide sequence corresponded to GenBank MT380725.1 [48]. The nucleotide sequence encoding RBD was amplified by PCR using primers F_RBD_319–524_SexAI (ATACCAGGTG GTGCAGCCCA CCGAATCCAT) and R_RBD_319–524_StuI (ATAGGCCTCA CTGTGGCAGG GGCATGCA). For cloning of RBD, we used the previously obtained vector pQE30_Flg4M2eHA2-2 encoding flagellin of *S. typhimurium* (GenBank ATU59429) carrying N-terminal hexahistidine tag and linked to influenza A virus peptides [49]. The sequence coding for these antigens was excised and replaced with the RBD sequence. Then the hybrid Flg-RBD gene was amplified by PCR and cloned into pEff at the AscI and SmaI sites. *E. coli* strains were grown in LB broth or on LB agar plates at 37 °C with kanamycin. The constructed recombinant vector pEff-Flg-RBD was transferred from *E. coli* to *A. tumefaciens* GV3101 using electroporation. Previously constructed recombinant vector pEff-Flg was used for the production of his-tagged empty flagellin [40].

### 4.3. Agroinfiltration of N. benthamiana Leaves

*N. benthamiana* plants were grown in a greenhouse in a 16 h daylight regime with additional illumination for about 6 weeks. The strain of *A. tumefaciens* GV3101 carrying the pEff-Flg-RBD (or pEff-Flg) vector was grown in LB broth with kanamycin (50 µg/mL), rifampicin (50 µg/mL), gentamycin (25 µg/mL) overnight at a 28 °C with shaking. The agrobacterial cells (10 mL) were collected by centrifugation at 4000× *g* for 5 min and the cells pellet was resuspended in 10 mL of a solution containing 10 mM MES (pH 5.5) and 10 mM MgSO_4_. A suspension of agrobacteria (OD_600nm_ ~ 0.2) was injected into the *N. benthamiana* leaves by a needleless syringe. After agroinfiltration, plants were further cultivated in a greenhouse under the same conditions.

### 4.4. SDS-PAGE and Western Blotting

Pieces of agroinfiltrated leaves (~10 mg) were excised, placed into 50 µL of the extraction buffer (50 mM Tris pH 8.0, 0.4 M sucrose, 5 mM MgCl_2_, 10% glycerol, 5 mM β-mercaptoethanol), and homogenized. To obtain soluble protein fraction, the mixture was subjected to centrifugation at 14,000× *g* for 10 min and the supernatant was collected. An equal volume of loading buffer for SDS-PAGE (20% glycerol, 5% SDS, 62.5 mM Tris pH 6.8, 0.5% bromphenol blue, 5% β-mercaptoethanol) was added to the initial suspension (total protein fraction) or the supernatant (soluble protein fraction). Then, 10 μL of the obtained solution (corresponding to about 1 mg of leaf biomass) was analyzed by SDS-PAGE (10%). After electrophoresis, the bands were visualized by staining of the gel with One-Step Blue Protein Gel Stain (BIOTIUM, Fremont, CA, USA).

For Western blotting, the proteins separated in the SDS-PAGE gel were transferred onto a Hybond-P membrane (GE Healthcare, New York, NY, USA) employing the Trans-Blot Turbo Transfer System (Bio-Rad Laboratories, Hercules, CA, USA). For the Western blotting with anti-Flg antibodies the membrane was blocked with a 5% (*w/v*) solution of dry milk in TBS-T (20 mM Tris pH 8.0, 150 mM NaCI, 0.1% Tween 20) buffer for 1 h at room temperature and subsequently incubated with mouse polyclonal anti-Flg antigen primary antibodies (used at a dilution of 1:1000) for 1 h at room temperature. Then the membrane was washed three times with TBS-T buffer (15 min at room temperature) and incubated with secondary rabbit anti-mouse antibodies conjugated with peroxidase (Promega, Madison, WI, USA) for 1 h at room temperature. For the Western blotting with antibodies against the hexahistidine tag (1 mg/mL, used at a dilution of 1:1000) and RBD (2 mg/mL, used at a dilution of 1:2000) we used iBind Western System (Invitrogen, Waltham, MA, USA) following the manufacturer’s instructions. The rabbit anti-mouse antibodies conjugated with peroxidase (Promega, Madison, WI, USA) were used as the secondary antibodies at a dilution of 1:10,000. Specific protein-antibody complexes were visualized using a Western Blot ECL Plus kit (GE Healthcare, New York, NY, USA) and chemiluminescence detector Fusion Solo X (Vilber, Eberhardzell, Germany).

Monoclonal antibodies against the hexahistidine tag (ab18184, Abcam), monoclonal antibodies against the RBD (Xema, Russia), and mouse polyclonal antibodies to the flagellin kindly provided by Dr. Liudmila Tsybalova from the Research Institute of Influenza (St. Petersburg, Russia) were used in Western blotting experiments.

The efficiency of Flg-RBD expression was estimated in Western blot analysis in comparison to purified Flg-RBD protein with anti-Flg antibodies (Supplementary File S3). The relative intensities of the bands in the digital photograph were measured using Nonlinear Dynamics TotalLab TL120 v2009 software.

### 4.5. Isolation of Recombinant Proteins from Plant Biomass and Purification

For large-scale isolation, the plant-produced Flg-RBD protein was purified on Ni-NTA resin (Promega, Madison, WI, USA) under denaturing conditions. Four days after infiltration, the *N. benthamiana* leaves were homogenized in a solution containing 6 M guanidine-HCI, 50 mM NaH_2_PO_4_, 500 mM NaCI (pH 8.0). The obtained mixture was subjected to centrifugation (14,000× *g* for 15 min). The supernatant was loaded onto Ni-NTA resin pre-incubated with the same buffer and allowed to stand for 60 min. Flow-through fraction of Flg-RBD was re-loaded onto Ni-NTA resin. The resin was subsequently washed with the buffers (8 M urea, 50 mM NaH_2_PO_4_, 300 mM NaCI) containing 10 mM and 20 mM imidazole. The recombinant protein Flg-RBD was eluted from the resin in a buffer with 4 M urea, 50 mM NaH_2_PO4, 300 mM NaCI and 500 mM imidazole.

After elution, the protein sample was dialyzed three times against PBS (1:100) using Slide-A-Lyzer Mini dialysis units (Thermo Fisher Scientific, Waltham, MA, USA). After dialysis, the protein sample was clarified by filtration through 0.22 µm Sprintzen/Syringe-Filter (TPP Techno Plastic Products AG, Trasadingen, Switzerland). Protein amounts were measured using a Qubit Protein Assay Kit using Qubit Fluorometer and relevant protocols (Invitrogen, Waltham, MA, USA). The protein preparation was stored at −20 °C. The control flagellin without RBD was produced in *N. benthamiana* and purified in the same way as Flg-RBD.

## 5. Conclusions

Overall, this study demonstrated that recombinant protein comprising Flg and the RBD of SARS-CoV-2 can be produced in plants up to 100 μg/g of fresh plant biomass using self-replicating viral vector pEff. Plant-produced Flg-RBD protein could be further used for the development of recombinant mucosal vaccines against COVID-19 that could be delivered intranasally. Induction of the immune response in the nasal compartment is particularly important for COVID-19 vaccines since it can prevent the establishment of infection in individuals and prevent the spread of the disease.

## Data Availability

Not applicable.

## Supplementary Materials

The following are available online at https://www.mdpi.com/article/10.3390/plants10122682/s1. Supplementary File S1: Western blot of proteins isolated from *N. benthamiana* plants infiltrated with pEff-Flg-RBD at various times. Supplementary File S2: Expression of RBD in *N. benthamiana*. Supplementary File S3: Determination of the efficiency of Flg-RBD expression in *N. benthamiana*.

## Abbreviations

AMV	5′-nontranslated region of RNA 4 of the alfalfa mosaic virus
ACE2	Angiotensin-converting enzyme 2
Flg	Flagellin
GFP	Green fluorescent protein
NTA	Nitrilotriacetic acid
PBS	Phosphate buffered saline
PVX	Potato virus X
RBD	Receptor Binding Domain of the S protein of SARS-CoV-2
SARS-CoV-2	Severe acute respiratory syndrome coronavirus 2
TLR	Toll-like receptor

## References

[1] WHO (World Health Organization). https://www.who.int/emergencies/diseases/novel-coronavirus-2019.

[2] Huang Y., Yang C., Xu X., Xu W., Liu S. (2020). Structural and functional properties of SARS-CoV-2 spike protein: Potential antivirus drug development for COVID-19. Acta Pharmacol. Sin..

[3] Walls A.C., Park Y.J., Tortorici M.A., Wall A., McGuire A.T., Veesler D. (2020). Structure, function, and antigenicity of the SARS-CoV-2 spike glycoprotein. Cell.

[4] Letko M., Marzi A., Munster V. (2020). Functional assessment of cell entry and receptor usage for SARS-CoV-2 and other lineage B betacoronaviruses. Nat. Microbiol..

[5] Watanabe Y., Allen J.D., Wrapp D., McLellan J.S., Crispin M. (2020). Site-specific glycan analysis of the SARS-CoV-2 spike. Science.

[6] Wrapp D., Wang N., Corbett K.S., Goldsmith J.A., Hsieh C.L., Abiona O., Graham B.S., McLellan J.S. (2020). Cryo-EM structure of the 2019-nCoV spike in the prefusion conformation. Science.

[7] Lan J., Ge J., Yu J., Shan S., Zhou H., Fan S., Zhang Q., Shi X., Wang Q., Zhang L. (2020). Structure of the SARS-CoV-2 spike receptor-binding domain bound to the ACE2 receptor. Nature.

[8] Dai L., Gao G.F. (2020). Viral targets for vaccines against COVID-19. Nat. Rev. Immunol..

[9] Lomonossoff G.P., D’Aoust M.-A. (2016). Plant-produced biopharmaceuticals: A case of technical developments driving clinical deployment. Science.

[10] Schillberg S., Raven N., Spiegel H., Rasche S., Buntru M. (2019). Critical Analysis of the Commercial Potential of Plants for the Production of Recombinant Proteins. Front. Plant Sci..

[11] Schillberg S., Finnern R. (2021). Plant molecular farming for the production of valuable proteins—Critical evaluation of achievements and future challenges. J. Plant Physiol..

[12] Wilson S.A., Roberts S.C. (2012). Recent advances towards development and commercialization of plant cell culture processes for the synthesis of biomolecules. Plant Biotechnol. J..

[13] Dixon C., Wilken L.R., Woodard S.L., Barros G.O.F. (2018). The Impact of Six Critical Impurities on Recombinant Protein Recovery and Purification from Plant Hosts. Molecular Pharming: Applications, Challenges, and Emerging Areas.

[14] Kusnadi A.R., Nikolov Z.L., Howard J.A. (1997). Production of recombinant proteins in transgenic plants: Practical considerations. Biotechnol. Bioeng..

[15] Thuenemann E.C., Lenzi P., Love A.J., Taliansky M., Becares M., Zuniga S., Enjuanes L., Zahmanova G.G., Minkov I.N., Matić S. (2013). The use of transient expression systems for the rapid production of virus-like particles in plants. Curr. Pharm. Des..

[16] Sainsbury F., Lomonossoff G.P. (2008). Extremely high-level and rapid transient protein production in plants without the use of viral replication. Plant Physiol..

[17] Giritch A., Marillonnet S., Engler C., Van Eldik G., Botterman J., Klimyuk V., Gleba Y. (2006). Rapid high-yield expression of full-size IgG antibodies in plants coinfected with noncompeting viral vectros. Proc. Natl. Acad. Sci. USA.

[18] Gleba Y., Klimyuk V., Marillonnet S. (2007). Viral vectors for the expression of proteins in plants. Curr. Opin. Biotechnol..

[19] Marillonnet S., Giritch A., Gils M., Kandzia R., Klimyuk V., Gleba Y. (2004). In planta engineering of viral RNA replicons: Efficient assembly by recombination of DNA modules delivered Agrobacterium. Proc. Natl. Acad. Sci. USA.

[20] Marillonnet S., Thoeringer C., Kandzia R., Klimyuk V., Gleba Y. (2005). Systemic Agrobacterium tumefaciens–mediated transfection of viral replicons for efficient transient expression in plants. Nat. Biotechnol..

[21] Lindbo J.A. (2007). TRBO: A High-Efficiency Tobacco Mosaic Virus RNA-Based Overexpression Vector. Plant Physiol..

[22] Yamamoto T., Hoshikawa K., Ezura K., Okazawa R., Fujita S., Takaoka M., Mason H.S., Ezura H., Miura K. (2018). Improvement of the transient expression system for production of recombinant proteins in plants. Sci. Rep..

[23] Castilho A., Windwarder M., Gattinger P., Mach L., Strasser R., Altmann F., Steinkellner H. (2014). Proteolytic and N-glycan processing of human α1-antitrypsin expressed in *Nicotiana benthamiana*. Plant Physiol..

[24] Zischewski J., Sack M., Fischer R. (2016). Overcoming low yields of plant-made antibodies by a protein engineering approach. Biotechnol. J..

[25] Capell T., Twyman R.M., Armario-Najera V., Ma J.K., Schillberg S., Christou P. (2020). Potential Applications of Plant Biotechnology against SARS-CoV-2. Trends Plant Sci..

[26] Dhama K., Natesan S., Iqbal Yatoo M., Patel S.K., Tiwari R., Saxena S.K., Harapan H. (2020). Plant-based vaccines and antibodies to combat COVID-19: Current status and prospects. Hum. Vaccines Immunother..

[27] Rosales-Mendoza S., Márquez-Escobar V.A., González-Ortega O., Nieto-Gómez R., Arévalo-Villalobos J.I. (2020). What Does Plant-Based Vaccine Technology Offer to the Fight against COVID-19. Vaccines.

[28] Ward B.J., Makarkov A., Séguin A., Pillet S., Trépanier S., Dhaliwall J., Libman M.D., Vesikari T., Landry N. (2020). Efficacy, immunogenicity, and safety of a plant-derived, quadrivalent, virus-like particle influenza vaccine in adults (18–64 years) and older adults (≥65 years): Two multicentre, randomised phase 3 trials. Lancet.

[29] Ward B.J., Gobeil P., Séguin A., Atkins J., Boulay I., Charbonneau P.Y., Couture M., D’Aoust M.A., Dhaliwall J., Finkle C. (2021). Phase 1 randomized trial of a plant-derived virus-like particle vaccine for COVID-19. Nat. Med..

[30] Peyret H., Steele J.F.C., Jung J.-W., Thuenemann E.C., Meshcheriakova Y., Lomonossoff G.P. (2021). Producing vaccines against enveloped viruses in plants: Making the impossible, difficult. Vaccines.

[31] Mett V., Musiychuk K., Bi H., Farrance C.E., Horsey A., Ugulava N., Shoji Y., de la Rosa P., Palmer G.A., Rabindran S. (2008). A plant-produced influenza subunit vaccine protects ferrets against virus challenge. Influenza Other Respir. Viruses.

[32] https://ibioinc.com/technologies/lickm/.

[33] Moyle P.M., Toth I. (2013). Modern subunit vaccines: Development, components, and research opportunities. ChemMedChem.

[34] Foged C., Hansen J., Agger E.M. (2012). License to kill: Formulation requirements for optimal priming of CD8(+) CTL responses with particulate vaccine delivery systems. Eur. J. Pharm. Sci..

[35] Kim M.Y., Copland A., Nayak K., Chandele A., Ahmed M.S., Zhang Q., Diogo G.R., Paul M.J., Hofmann S., Yang M.S. (2018). Plant-expressed Fc-fusion protein tetravalent dengue vaccine with inherent adjuvant properties. Plant Biotechnol. J..

[36] Kumar H., Kawai T., Akira S. (2011). Pathogen recognition by the innate immune system. Int. Rev. Immunol..

[37] McDonald W.F., Huleatt J.W., Foellmer H.G., Hewitt D., Tang J., Desai P., Price A., Jacobs A., Takahashi V.N., Yan Huang N.V. (2007). A West Nile Virus Recombinant Protein Vaccine That Coactivates Innate and Adaptive Immunity. J. Infect Dis..

[38] Huleatt J.W., Nakaar V., Desai P., Huang Y., Hewitt D., Jacobs A., Tang J., McDonald W., Song L., Evans R.K. (2008). Potent immunogenicity and efficacy of a universal influenza vaccine candidate comprising a recombinant fusion protein linking influenza M2e to the TLR5 ligand flagellin. Vaccine.

[39] Stepanova L.A., Kotlyarov R.Y., Kovaleva A.A., Potapchuk M.V., Korotkov A.V., Sergeeva M., Kasianenko M.A., Kuprianov V.V., Ravin N.V., Tsybalova L.M. (2015). Protection against Multiple Influenza A Virus Strains Induced by Candidate Recombinant Vaccine Based on Heterologous M2e Peptides Linked to Flagellin. PLoS ONE.

[40] Blokhina E.A., Mardanova E.S., Stepanova L.A., Tsybalova L.M., Ravin N.V. (2020). Plant-Produced Recombinant Influenza A Virus Candidate Vaccine Based on Flagellin Linked to Conservative Fragments of M2 Protein and Hemagglutintin. Plants.

[41] Mardanova E.S., Kotlyarov R.Y., Kuprianov V.V., Stepanova L.A., Tsybalova L.M., Lomonosoff G.P., Ravin N.V. (2015). Rapid high-yield expression of a candidate influenza vaccine based on the ectodomain of M2 protein linked to flagellin in plants using viral vectors. BMC Biotechnol..

[42] Rattanapisit K., Shanmugaraj B., Manopwisedjaroen S., Purwono P.B., Siriwattananon K., Khorattanakulchai N., Hanittinan O., Boonyayothin W., Thitithanyanont A., Smith D.R. (2020). Rapid production of SARS-CoV-2 receptor binding domain (RBD) and spike specific monoclonal antibody CR3022 in *Nicotiana benthamiana*. Sci. Rep..

[43] Mamedov T., Yuksel D., Ilgın M., Gürbüzaslan I., Gulec B., Mammadova G., Say D., Hasanova G. (2020). Engineering, production and characterization of Spike and Nucleocapsid structural proteins of SARS–CoV-2 in *Nicotiana benthamiana* as vaccine candidates against COVID-19. bioRxiv.

[44] Maharjan P.M., Cheon J., Jung J., Kim H., Lee J., Song M., Jeong G.U., Kwon Y., Shim B., Cho S. (2021). Plant-Expressed Receptor Binding Domain of the SARS-CoV-2 Spike Protein Elicits Humoral Immunity in Mice. Vaccines.

[45] Mardanova E.S., Blokhina E.A., Tsybalova L.M., Peyret H., Lomonossoff G.P., Ravin N.V. (2017). Efficient Transient Expression of Recombinant Proteins in Plants by the Novel pEff Vector Based on the Genome of Potato Virus, X. Front. Plant Sci..

[46] Thuenemann E.C., Byrne M.J., Peyret H., Saunders K., Castells-Graells R., Ferriol I., Santoni M., Steele J.F.C., Ranson N.A., Avesani L. (2021). A Replicating Viral Vector Greatly Enhances Accumulation of Helical Virus-Like Particles in Plants. Viruses.

[47] Takova K., Koynarski T., Minkov G., Toneva V., Mardanova E., Ravin N., Lukov G.L., Zahmanova G. (2021). Development and Optimization of an Enzyme Immunoassay to Detect Serum Antibodies against the Hepatitis E Virus in Pigs, Using Plant-Derived ORF2 Recombinant Protein. Vaccines.

[48] Amanat F., Stadlbauer D., Strohmeier S., Nguyen T.H.O., Krammer F. (2020). A serological assay to detect SARS-CoV-2 seroconversion in humans. Nat. Med..

[49] Blokhina E.A., Mardanova E.S., Tsybalova L.M., Ravin N.V. (2018). Expression in Plants of a Recombinant Protein Based on Flagellin Linked to Conservative Fragments of M2 Protein and Hemagglutintin of Influenza Virus. Appl. Biochem. Microbiol..

