# Supplementary material for: High-Yield Production of Receptor Binding Domain of SARS-CoV-2 Linked to Bacterial Flagellin in Plants Using Self-Replicating Viral Vector pEff
Source: Plants (Basel). 2021 Dec 6;10(12):2682. doi: 10.3390/plants10122682 (PMC8708900; doi:10.3390/plants10122682)
Supplement: Supplementary file 1 [file plants-10-02682-s001.zip › Supplementary file 3_rev.pdf]

Supplementary File 3

Determination of the efficiency of Flg-RBD expression in *N. benthamiana*

The efficiency of Flg-RBD expression was estimated in Western blot analysis in comparison to known amount of purified Flg-RBD protein. The amount of purified Flg-RBD was measured using a Qubit Protein Assay Kit (Invitrogen, USA).

Western blot probed with antibodies against Flg

1 2 3 4 5 6 7

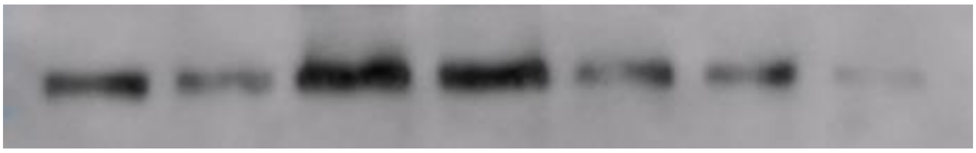

Lanes 1 and 2, – total proteins from 3 mg and 1.5 mg of leaf tissue of *N. benthamiana* infiltrated with pEff-Flg-RBD vector, respectively

Lanes 3-7, - 0.76 μg, 0.57 μg. 0.38 μg, 0.19 μg and 0.095 μg of purified Flg-RBD protein, respectively

Densitometry analysis of Western blot image.

The digital image was subjected to densitometry analysis using Nonlinear Dynamics TotalLab TL120 v2009 software.

	Lane 1	Lane 2	Lane 3
Densitometry curve	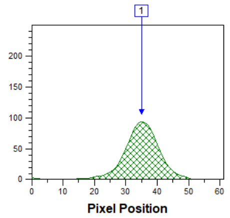	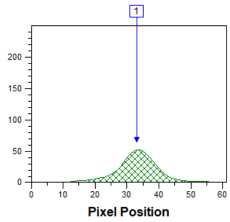	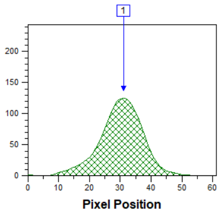
Band intensity (relative units)	62894	36328	108138

Lane 4	Lane 5	Lane 6	Lane 7
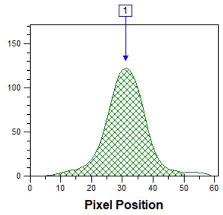	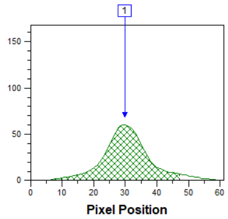	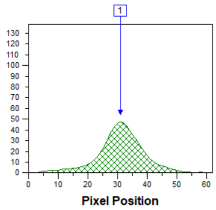	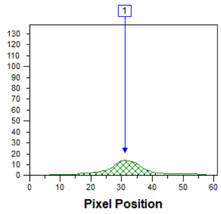
96921	55946	37504	9164

Calculation of expression efficiency

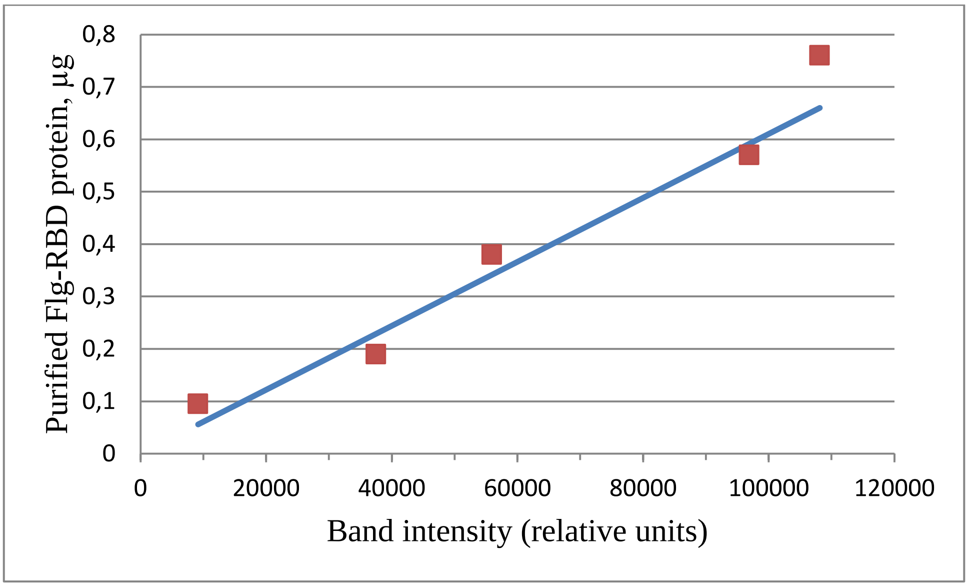

The efficiency of expression = 135 µg per 1 g of leaf tissue.
