# Supplementary material for: High-Yield Production of Receptor Binding Domain of SARS-CoV-2 Linked to Bacterial Flagellin in Plants Using Self-Replicating Viral Vector pEff
Source: Plants (Basel). 2021 Dec 6;10(12):2682. doi: 10.3390/plants10122682 (PMC8708900; doi:10.3390/plants10122682)
Supplement: Supplementary file 1 [file plants-10-02682-s001.zip › Supplementary file 1.pdf]

Supplementary File 1

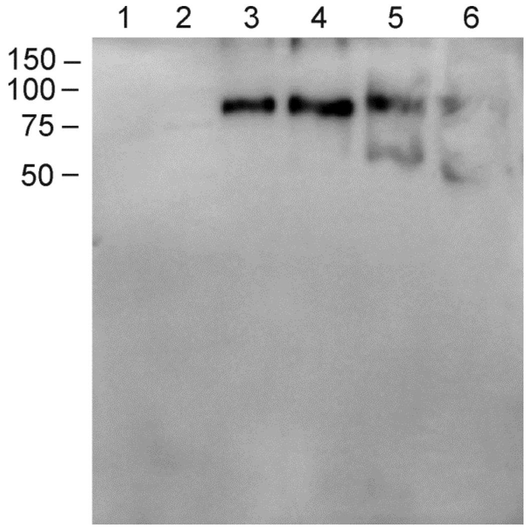

Figure S1. Western blot of proteins isolated from *N. benthamiana* plants infiltrated with pEff-Flg-RBD on days 3, 4, 5 and 6 after infiltration.
Lane 1, total proteins isolated from non-infiltrated leaf;
Lane 2, molecular weight marker (sizes are shown in kD);
Lanes 3-6, total protein isolated from leaf infiltrated with pEff-Flg-RBD on days 3, 4, 5 and 6 after infiltration
Western blotting was performed using anti-flagellin antibodies.
