# Supplementary material for: High-Yield Production of Receptor Binding Domain of SARS-CoV-2 Linked to Bacterial Flagellin in Plants Using Self-Replicating Viral Vector pEff
Source: Plants (Basel). 2021 Dec 6;10(12):2682. doi: 10.3390/plants10122682 (PMC8708900; doi:10.3390/plants10122682)
Supplement: Supplementary file 1 [file plants-10-02682-s001.zip › Supplementary file 2.pdf]

Supplementary File 2

Expression of RBD in *N. benthamiana*

To obtain the viral vector pEff-RBD allowing expression of the RBD protein, the corresponding gene was cloned in the pEff vector. Vector pEff-RBD was transfected into *A. tumefaciens* GV3101 and the resulting agrobacterium cultures were used for the infiltration of the leaves of *N. benthamiana*. Leaf tissues for isolation of protein samples were harvested on day 4 after agroinfiltration since in the following days necrosis of leaf tissues was observed.

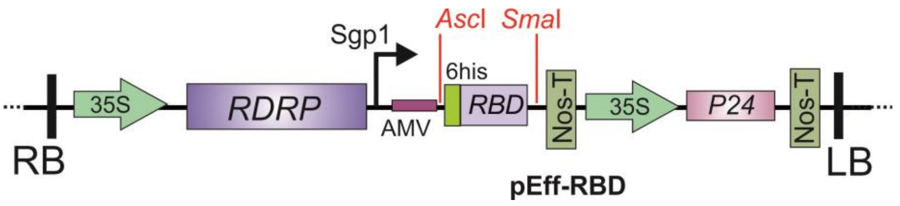

Due to low expression level we unable to detect RBD in SDS-PAGE and Western blotting. The protein was purified from infiltrated leaf tissues using metal-affinity chromatography under denaturing conditions. The SDS-PAGE analysis of purified proteins showed that the RBD protein was isolated but appeared to be heavily contaminated by RuBisCo.

The amount of purified proteins was measured using a Qubit Protein Assay Kit (Invitrogen, USA). The share of RBD was estimated from densitometry analysis of digital SDS-PAGE image using Nonlinear Dynamics TotalLab TL120 v2009 software. RBD accounted for 16.3% of the total 77.9 μg protein isolated from 1 mg of biomass.

The yield of RBD is therefore about 13 μg per 1 g of leaf tissue.

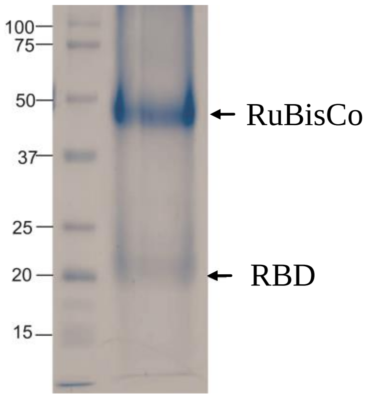

SDS-PAGE of partly purified RBD protein

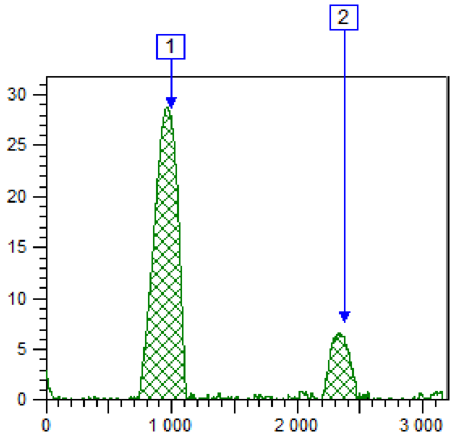

(1) RuBisCo band intensity 4,907,037 relative units
(2) RBD band intensity 954,591 relative units
